# The Treatment of Meningitis of Otitic Origin

**Published:** 1913-01-25

**Authors:** 


### THE TREATMENT OF MENINGITIS OF OTITIC ORIGIN.

### THE ROYAL SOCIETY OF MEDICINE.

On Friday, 17th inst., the meeting of the Otological Section, Royal Society of Medicine, discussed the subject of Meningitis. The debate was introduced in a very practical paper by Dr. William Milligan, of Manchester, who remarked that of the various intracranial complications consecutive to ear disease none were, at times, more difficult to diagnose or more disappointing in treatment. Yet he believed that surgery, which had gained so many victories over the army of septic organisms, would be able in the future to cope with this disease, and thus add one more to its many laurels. Meningitis serosa was indicated clinically by headache, vomiting, stiffness of neck muscles, and increase of arterial tension. There would always be inherent differences in the clinical landscapes, due to the variety and toxicity of the causal organisms and the cell-resistance of the patient. The disease was not necessarily fatal provided an early diagnosis was made and prompt treatment instituted, especially early drainage. Courage was needed to operate when there was an absence of the full ensemble of text-book symptoms. Practically, meningitis was divisible into localised and diffuse. The most formidable variety was purulent leptomeningitis, *i.e.* when the meshes of the pia arachnoid were involved; it was frequently encountered with brain abscess or sinus phlebitis. But the fact that for a time it remained localised to the original focus imparted some hope to the surgeon. He believed that accessions of temperature indicated that fresh areas of tissue were being involved.

When all the text-book symptoms were present the patient was usually beyond surgical salvation. So long as it remained able to deal with bacterial attacks, the cerebro-spinal fluid was alkaline and more or less clear. But increased turbidity did not necessarily mean that it had become septic, but that the lymphocytosis resulted from the supreme effort to ward off invasion. To attain the victory, cerebro-spinal fluid had to be secreted in such quantity that the patient might become almost comatose from the increased intracranial tension. If at this stage the infecting focus be removed, recovery not infrequently ensued. The copper-reducing test of Kopetzky was an easy, and apparently certain, means of early diagnosis. A diminishing alkalinity, or definite acidity of the fluid, was another useful bedside indication of what was taking place. Should the fluid have become acid and very turbid a good result from operation is rendered less likely. Films or cultures did not reveal bacteria in the early stages of the disease. Increase of intracranial tension was accompanied by heightened blood pressure and a tendency to cerebral anæmia. At this stage lumbar puncture was a useful therapeutic proceeding.

Another indication of commencing meningitis was œdema or fluffiness of the edge of the optic disc. Ventricular drainage carried the risk of infecting a previously non-infected ventricle, as well as the cerebral substance through which the needle had passed. Urotropine given internally was said to have a beneficial effect in keeping the cerebro-spinal fluid aseptic. Where the labyrinth was the primary focus of infection he recommended a decompression operation in the posterior fossa, at some distance from the original focus, so as to avoid risk of additional infection. The internal ear he regarded as the most frequent and dangerous avenue of infection to the meninges, because it led direct to the posterior fossa. After removal of the focus, the surgeon's efforts should be directed to the relief of intracranial pressure and the maintenance of free drainage from the meninges.

Dr. Milligan then discussed the technique of operations devised to prevent herniation of the brain substance, and stated that his own cases showed thirty-seven cases of meningitis serosa with eight fatalities; in all the fatal cases the fluid had become definitely purulent, and operation proved unavailing.

The subsequent debate, mainly on questions of technique, was taken part in by the President (Dr. Dundas Grant), Mr. C. E. West, Mr. Sydney Scott, and Dr. Dan McKenzie. Dr. Milligan was heartily thanked for his paper, and replied in detail.

